# Supplementary material for: A high-quality reference genome for the Ural Owl (Strix uralensis) enables investigations of cell cultures as a genomic resource for endangered species
Source: Gigascience. 2025 Sep 23;14:giaf106. doi: 10.1093/gigascience/giaf106 (PMC12455985; doi:10.1093/gigascience/giaf106)
Supplement: giaf106_Authors_Response_To_Reviewer_Comments_Original_Submission [file giaf106_authors_response_to_reviewer_comments_original_submission.pdf]

Author's Response To Reviewer Comments

Reply to Reviewers
We thank the reviewers for their valuable suggestions and provide point-by-point replies to the questions they raised below.
Reviewer 1

The authors provide a high-quality reference genome for the Ural Owl (Strix uralensis), these genomic resources are valuable for conservation and evolution. The manuscript is well-written, and the scientific story with cell culture for conservation is interesting.
We thank Dr Jian for the positive evaluation of our work.

I have some questions or comments as following:
1. in abstract， the N50 is contig or scaffold？
We refer to scaffold N50 and have added this information to the abstract.

2. For the GenomeScope analysis, the estimated genome size is 1.29 Gb with low heterozygosity (0.2%). The assembled genome size is 1.38 Gb. Could there be duplicated genome sequences in the assembly, or did the genome survey evaluation exclude some k-mers? What were the parameters used in GenomeScope2 (e.g., was the -h parameter set to its default value)?
We have added the use of default parameters in GenomeScope2 to the corresponding method section. GenomeScope delivers an estimate of the genome size. The value we obtained was in the expected range. Yet, GenomeScope often underestimates genome size compared to obtained assemblies or other genome size estimation methods (e.g. Pflug et al. 2020 G3, Natarajan et al 2025 BMC Genomics). We further refer the reviewer to supplementary table 1 for a comparison of all primary and alternate assembly sizes to show that the assembly used had the most equal split of all of our runs. We suspect a combination of low frequency kmers resulting from the high repeat content to cause the GenomeScope underestimation.
Line 565: "GenomeScope2 v2.0.1 [68] with default parameters was used to estimate genome size, heterozygosity, and the homozygous and heterozygous coverage peaks. "

3. How do you perform the decontamination？
Decontamination was performed using Kraken2 v2.1.3. This information is included in the methods description.
Line 554 "Contaminant sequences were filtered from the HiFi reads using Kraken2 v2.1.3 [63,64] with the Kraken database kraken2 PlusPFP downloaded in March 2023 and parameters "-- confidence 0.51 --use-names".
Line 574 "To remove contamination from the short, Hi-C reads, Kraken2 v2.1.3 was used similarly to the HiFi reads but in paired-read mode, with parameter "--paired".
Line 732: ".. with Kraken2 v2.1.3 with the Kraken database kraken2 PlusPFP database downloaded in March 2023 in paired-read mode, with parameter "--paired --confidence 0.51 --use-names"

4. For the Hi-C contact map, due to some chromosomes are considerably larger while others are much smaller, it is suggested that the larger chromosomes could be displayed independently from the smaller ones to enhance clarity and interpretation.
We now provide a HiC contact map visualization of the 10 largest chromosomes in the main text (Figure 5), the previous version of this figure and a version with chromosomes 11 to 41 are now included in the supplement (Supplementary Figures S2 and S3).

Reviewer 2

This manuscript provides a high-quality genome of Ural owl which is of evolutionary and ecological importance, as well as cell cultures that is worth exploration for endangered species. But Oral owl does not seem to be an endangered species?
We thank Dr. Xu for his positive feedback. We have extended the information on the species status in the

introduction (lines 82-96). The reviewer is right that on the global scale IUCN red list, the Ural Owl is classified as “Least Concern”. However, the species went completely extinct in Germany and Austria and hence was listed “extinct” in their respective national red lists. Reintroduction programs are currently in place in both countries. The Ural Owl is further a species of conservation measures throughout the European Union under the EU Birds Directive and Nature Habitats Directive. It is also part of the Bern Convention (Convention on the Conservation of European Wildlife and Nature Habitats). International trade of the species is regulated over Appendix II of CITES. CITES Appendix II covers species that are not necessarily now threatened with extinction but that may become so unless trade is closely controlled. We thus think that the Ural Owl is indeed an endangered species.

One chromosomal fusion was identified, but it is very important to specific which chromosome. The chromosomes are very conserved in birds. The authors should follow the chromosome nomenclature according to chicken chromosome homology (
We have added syntenic information to the chicken and zebra finch genomes (Figure 9) and provide a table (Supplementary Table S6) summarizing the synteny results allowing easy access to chromosomal projections across assemblies. The fusion we detected corresponds to Snowy Owl chromosomes 5 and 6 (as named in Baalsrud et al. 2024), which are syntenic to chicken chromosomes 4 and 5.

"bird microchromosomes have a higher density of genes" is already known for 20 years, so no need to confirm again.
We have moved the findings of this section to the supplement but would like to keep their confirmation with our high-quality assembly.

It is very speculative to link karatin gene expansions to flight adaptions. I suggest to revise this statement throughout the manuscript.
We have down-toned our statements; however given the findings from other studies on the importance of keratins in wing development and function, we would argue to keep our matching results in the text. We have consistent findings for the common ancestor of Accipitriformes and Strigiformes and for the last common ancestor of Strigiformes. The role of keratin genes in feather development and function has been documented before (Alibardi and Toni 2008, Greenwold et al. 2014, Pan et al. 2019, etc.) but asks for special attention in birds with novel flight adaptations. We hence think a focus on the keratin gene family expansion findings is justified and further substantiated by the fact that the total number of gene gains we found is relatively small.

The first paragraph lacks any citations. And the statements are not fully accurate because there are already reference genomes in Strigiformes (owls), some of which were generated by the bioEarch project.
We have added references to this section. The point we wanted to make here is that there are indeed, as the reviewer points out, reference genomes for owls but that even in a group as well studied as birds, gold standard T2T genome assemblies are rather the exception than the rule. We have modified the text to make this clearer.

L120, I don't think this is true?
We have carefully checked all publicly available bird genomes on the date of our manuscript submission and came to the conclusion that as of then, our assembly had the highest BUSCO score. In the INSDC records of reference genomes for birds, Pseudopodoces humilis is reported as having the highest BUSCO score with 99.8. Using compleasm, our Ural Owl assembly’s gene completeness scores at 99.94. We have edited the sentence in question.

L131, remove million?
The sentence has been corrected.

L158, again, the authors need to make sure that those chromosomes are homologous to chicken chromosomes. It is known that the 10 smallest microchromosomes are difficult for assembly due to HiFi sequencing dropout (Huang 2023 PNAS). I am curious whether the 10 smallest microchromosomes (or dot chromosomes) have been correctly assembled? The figure 3 does not seem to show this information.
We have added a synteny comparison of the chromosomes 1-41 of our assembly with the Huang et al. 2023 chicken assembly (Figure 9, Supplementary Table S6).

For the 17 lost genes, are they lost in all reference genomes, or just "supported by more than one reference genome" (L260)?
The ancestors' sequence contents are reconstructed from all present-day genomes in that clade meaning that if an ancestral node appears to have lost 17 genes compared to their own ancestor those losses should be supported by all derived species.

It is not surprising to me that kerain, immune and olfactory receptor genes are independently expanded in different bird lineages.
We agree with the reviewer and do not state that this finding is surprising.

L284-285, this statement is not true, because females also have a Z chromosome. Maybe the sequence coverage of the Z chromosome can be used to confirm the sex.
The sentence in question has been changed.

L361, cite B10K publications.
We had included the URL to B10K, we have now added Zhang et al. 2014 and Zhang 2015.

L370, "identified" should be "confirmed"?
The sentence has been modified.

L378, this is a bit misleading, because it is clear that barn owls have microchromosomes.
Barn owls have chromosomes of a homogenous size that cannot as clearly be separated into micro- and macrochromosomes as for typical avian genomes. We would like to refer the reviewer to the literature we had cited here (Rebholz et al. 1993, Figure 15). We have also changed the text to better convey the information.

L382, "mainly composed of centromeric satellite DNA", and L387-388 are not true. LINEs the LTRs should still be the major repeat contents.
We would like to refer the reviewer to the publication we cited here, Baalsrud et al. 2024, which states "We find that the repeat DNA content in the relatively large snowy owl genome (1.6 Gb) is among the highest reported for any bird genome to date (28.34% compared to an average of ~10% in other birds). The bulk of the snowy owl genomic repeat landscape consists of centromeric satellite DNA, which appears to have originated from an endogenous retrovirus (ERV1). Using gene collinearity analyses we show that the position of these evolutionary new centromeres (ECNs) are not homologous with chicken centromeres, and are located in regions with collinearity breaks to other bird genomes due to chromosomal rearrangements. Our results support rapid transposable element-driven evolution of lineage-specific centromeres". We thus politely disagree with the reviewer and would like to leave the sentence unchanged. If the reviewer has data that would prove Baalsrud et al. wrong we would be happy to learn about them.

L395-396, "In birds, microchromosomes possibly originate from chromosome fission.", this is not true, again see Huang 2023 PNAS.
The sentence has been removed.

The paragraph starting from L394 is already well know. No need to discuss this. Overall, the discussion part needs to be streamlined, including the paragraph at L434 and L455
We have removed lines 394-398.
We have revised the other two paragraphs but see a need to include them since species conservation was a major motivation for our study. We have already been contacted by two breeding programs that highly appreciate the here generated resource.

Editorial Manager®

Cookie Preference Center

We use cookies which are necessary to make our site work. We may also use additional cookies to analyze, improve and personalize our content and your digital experience. For more information, see our Cookie Policy.

You may choose not to allow some types of cookies. However, blocking some types may impact your experience of our site and the services we are able to offer. See the different category headings below to find out more or change your settings.

Allow all

Manage Consent Preferences

Strictly Necessary Cookies

Always active

These cookies are necessary for the website to function and cannot be switched off in our systems. They are usually only set in response to actions made by you which amount to a request for services, such as setting your privacy preferences, logging in or filling in forms. You can set your browser to block or alert you about these cookies, but some parts of the site will not then work.

Functional Cookies

These cookies enable the website to provide enhanced functionality and personalisation. They may be set by us or by third party providers whose services we have added to our pages. If you do not allow these cookies then some or all of these services may not function properly.

Performance Cookies

These cookies allow us to count visits and traffic sources so we can measure and improve the performance of our site. They help us to know which pages are the most and least popular and see how visitors move around the site.

Targeting Cookies

These cookies may be set through our site by our advertising partners. They may be used by those companies to build a profile of your interests and show you relevant adverts on other sites. If you do not allow these cookies, you will experience less targeted advertising.

Cookie List

Clear

Apply Cancel
Consent Leg.Interest

Confirm my choices

Powered by onetrus
