# Supplementary material for: A high-quality reference genome for the Ural Owl (Strix uralensis) enables investigations of cell cultures as a genomic resource for endangered species
Source: Gigascience. 2025 Sep 23;14:giaf106. doi: 10.1093/gigascience/giaf106 (PMC12455985; doi:10.1093/gigascience/giaf106)
Supplement: giaf106_Authors_Response_To_Reviewer_Comments_Revision_1 [file giaf106_authors_response_to_reviewer_comments_revision_1.pdf]

# Author's Response To Reviewer Comments

Reply to Reviewers

Reviewer 1 - Jainbo Jian

The response is satisfactory. I suggest accepting the manuscript.
We thank Dr Jian for the efforts and positive evaluation of our work.

Reviewer 2 - Luohao Xu

L59, I believe most bird lineages lack golden standard T2T genomes except for Galliformes. In fact, the Ural Owl genome is also not a T2T assembly. Therefore, the writing here is potentially misleading.
We have changed the sentence in question to "However, even in genomically and traditionally well-studied groups such as birds, most lineages still lack high-quality reference genome assemblies that would allow for detailed studies of genome evolution."

Many figures can go to supplements, or merge into a larger figure.
We have moved previous figures 4, 8, 12, and 13 to the supplement, now figures S2, S8, S9 and S10 respectively.

Please add accession ID for chicken and zebra finch genomes.
We have added the information to lines 673-676 "... to estimate chromosome-scale alignments between G. gallus (GCA_024206055.2), T. guttata (GCF_048771995.1), S. aluco (GCA_031877795.1), S. uralensis, and B. scandiacus (GCA_965212795.1)."

According to Figure 9 and Supplementary table S6, some microchromosomes do not have clear synteny, in contrast to the Fig. 1C of https://www.nature.com/articles/s42003-023-05137-x. Perhaps add some discussion about this?
We thank the reviewer for pointing us to this publication. We have revised our synteny analysis, which now also shows the conserved synteny between chicken and zebrafinch chromosomes. We have further added information to the difference between owls and chicken/zebrafinch. While some microchromosomes indeed lack strong syntenic relationships to chicken/zebrafinch, they are conserved within owls. We now include this information in the revised manuscript. Still existing differences between our analyses and the one in Lou et al. can be explained by the usage of different synteny reconstructions, Luo et al. used MCscan, which is gene based whereas we used genome wide alignments.
We have added the following to the result section:
Lines 275-288 "Compared to chicken and zebrafinch however, we identified several large scale changes. We detected a fusion of chromosomes 5 and 6 of the Snowy Owl (corresponding to parts of chicken chromosome 4 and chromosome 5 and zebrafinch chromosome 4 and parts of 5, Figure 7 and Supplementary Table S6) into chromosome 4 of the two Strix assemblies. This is supported by previous cytogenetic analyses [27]. The remaining part of chicken chromosome 4 corresponds to Ural Owl chromosome 13, Snowy Owl chromosome 12 and zebrafinch 4a. A part of chicken and zebrafinch chromosome 5 corresponds to two chromosomes in the Ural Owl (chromosomes 16 and 30). For the remaining chromosomes of our Ural Owl assembly, we could mostly identify 1:1 relationships with chromosomes of Snowy Owl, chicken and zebrafinch with the exception of the Ural Owl microchromosomes 31, 35, 39 and 41 for which we could not unambiguously identify a corresponding chicken or zebrafinch chromosome but homology to Snowy Owl scaffolds and Tawny Owl chromosomes."
And the following to the discussion section:
Lines 370-376 "Besides this, we identified several other chromosomal rearrangements between owl genomes (which are overall very syntenic) and those of chicken and zebrafinch. For four microchromosomes we could not identify a reliable homologous chromosome in chicken nor zebrafinch yet syntenic scaffolds in the Snowy Owl and microchromosomes in the Tawny Owl assemblies. In addition, the

microchromosomes we assembled were also well supported by our HiC data. We thus hypothesize that the lack of synteny to chicken and zebrafinch might reflect more structural changes specific to owls.”

## Cookie Preference Center

We use cookies which are necessary to make our site work. We may also use additional cookies to analyze, improve and personalize our content and your digital experience. For more information, see our Cookie Policy.

You may choose not to allow some types of cookies. However, blocking some types may impact your experience of our site and the services we are able to offer. See the different category headings below to find out more or change your settings.

Allow all

## Manage Consent Preferences

### Strictly Necessary Cookies

**Always active**

These cookies are necessary for the website to function and cannot be switched off in our systems. They are usually only set in response to actions made by you which amount to a request for services, such as setting your privacy preferences, logging in or filling in forms. You can set your browser to block or alert you about these cookies, but some parts of the site will not then work.

### Functional Cookies

These cookies enable the website to provide enhanced functionality and personalisation. They may be set by us or by third party providers whose services we have added to our pages. If you do not allow these cookies then some or all of these services may not function properly.

### Performance Cookies

These cookies allow us to count visits and traffic sources so we can measure and improve the performance of our site. They help us to know which pages are the most and least popular and see how visitors move around the site.

**Targeting Cookies**

These cookies may be set through our site by our advertising partners. They may be used by those companies to build a profile of your interests and show you relevant adverts on other sites. If you do not allow these cookies, you will experience less targeted advertising.

**Cookie List**

Clear

Apply Cancel
Consent Leg.Interest

Confirm my choices

Powered by onetrus
