# Supplementary material for: A high-quality reference genome for the Ural Owl (Strix uralensis) enables investigations of cell cultures as a genomic resource for endangered species
Source: Gigascience. 2025 Sep 23;14:giaf106. doi: 10.1093/gigascience/giaf106 (PMC12455985; doi:10.1093/gigascience/giaf106)
Supplement: giaf106_Reviewer_2_Report_Original_Submission — Luohao Xu -- 5/5/2025 [file giaf106_reviewer_2_report_original_submission.pdf]

Reviewer Report

Title: A high-quality reference genome for the Ural Owl (Strix uralensis) enables investigations of cell cultures as a genomic resource for endangered species

Version: Original Submission **Date:** 5/5/2025

Reviewer name: Luohao Xu

Reviewer Comments to Author:

This manuscript provides a high-quality genome of Ural owl which is of evolutionary and ecological importance, as well as cell cultures that is worth exploration for endangered species. But Oral owl does not seem to be an endangered species?
One chromosomal fusion was identified, but it is very important to specific which chromosome. The chromosomes are very conserved in birds. The authors should follow the chromosome nomenclature according to chicken chromosome homology (http://pnas.org/doi/10.1073/pnas.2216641120).
"bird microchromosomes have a higher density of genes" is already known for 20 years, so no need to confirm again.
It is very speculative to link karatin gene expansions to flight adaptions. I suggest to revise this statement throughout the manuscript.
The first paragraph lacks any citations. And the statements are not fully accurate because there are already reference genomes in Strigiformes (owls), some of which were generated by the bioEarch project.
L120, I don't think this is true?
L131, remove million?
L158, again, the authors need to make sure that those chromosomes are homologous to chicken chromosomes. It is known that the 10 smallest microchromosomes are difficult for assembly due to HiFi sequencing dropout (Huang 2023 PNAS). I am curious whether the 10 smallest microchromosomes (or dot chromosomes) have been correctly assembled? The figure 3 does not seem to show this information.
For the 17 lost genes, are they lost in all reference genomes, or just "supported by more than one reference genome" (L260)?
It is not surprising to me that kerain, immune and olfactory receptor genes are independently expanded in different bird lineages.
L284-285, this statement is not true, because females also have a Z chromosome. Maybe the sequence coverage of the Z chromosome can be used to confirm the sex.
L361, cite B10K publications.
L370, "identified" should be "confirmed"?
L378, this is a bit misleading, because it is clear that barn owls have microchromosomes.
L382, "mainly composed of centromeric satellite DNA", and L387-388 are not true. LINEs the LTRs should still be the major repeat contents.
L395-396, "In birds, microchromosomes possibly originate from chromosome fission.", this is not true,

again see Huang 2023 PNAS.
The paragraph starting from L394 is already well know. No need to discuss this. Overall, the discussion part needs to be streamlined, including the paragraph at L434 and L455

Methods

Are the methods appropriate to the aims of the study, are they well described, and are necessary controls included? Choose an item.

Conclusions

Are the conclusions adequately supported by the data shown? Choose an item.

Reporting Standards

Does the manuscript adhere to the journal's guidelines on minimum standards of reporting? Choose an item.

Choose an item.

Statistics

Are you able to assess all statistics in the manuscript, including the appropriateness of statistical tests used? Choose an item.

Quality of Written English

Please indicate the quality of language in the manuscript: Choose an item.

Declaration of Competing Interests

Please complete a declaration of competing interests, considering the following questions:

- Have you in the past five years received reimbursements, fees, funding, or salary from an organisation that may in any way gain or lose financially from the publication of this manuscript, either now or in the future?
- Do you hold any stocks or shares in an organisation that may in any way gain or lose financially from the publication of this manuscript, either now or in the future?
- Do you hold or are you currently applying for any patents relating to the content of the manuscript?
- Have you received reimbursements, fees, funding, or salary from an organization that holds or has applied for patents relating to the content of the manuscript?
- Do you have any other financial competing interests?
- Do you have any non-financial competing interests in relation to this paper?

If you can answer no to all of the above, write 'I declare that I have no competing interests' below. If your reply is yes to any, please give details below.

I declare that I have no competing interests

I agree to the open peer review policy of the journal. I understand that my name will be included on my report to the authors and, if the manuscript is accepted for publication, my named report including any attachments I upload will be posted on the website along with the authors' responses. I agree for my report to be made available under an Open Access Creative Commons CC-BY license (http://creativecommons.org/licenses/by/4.0/). I understand that any comments which I do not wish to be included in my named report can be included as confidential comments to the editors, which will not be published.

Choose an item.

To further support our reviewers, we have joined with Publons, where you can gain additional credit to further highlight your hard work (see: https://publons.com/journal/530/gigascience). On publication of this paper, your review will be automatically added to Publons, you can then choose whether or not to claim your Publons credit. I understand this statement.

Yes Choose an item.
